# A ridge-loaded staggered double-vane slow wave structure for terahertz radiation sources

**DOI:** 10.1038/s41598-024-82796-8

**Published:** 2024-12-28

**Authors:** Jibran Latif, Zhanliang Wang, Atif Jameel, Bilawal Ali, Muhammad Khawar Nadeem, Yubin Gong

**Affiliations:** School of Electronic Science and Engineering, University of Electronic Science and Technology of China, Chengdu, China

**Keywords:** Electrical and electronic engineering, Electronics, photonics and device physics, Plasma physics

## Abstract

A ridge-loaded staggered double-vane slow-wave structure is proposed for terahertz radiation sources employing a sheet electron beam. This slow-wave structure has the advantages of enhanced electric field and energy density distribution and improved interaction impedance in the beam-wave interaction region. High-frequency characteristics are investigated for the proposed slow wave structure and compared with those of the staggered double-vane slow wave structure. The slow wave structure is fabricated and experimentally tested for transmission and reflection properties, revealing $S_{21}$ above -2 dB and $S_{11}$ below -17 dB at 0.34 THz for a backward wave oscillator. Steady transmission of the 21.7 kV sheet electron beam is achieved by designing a periodic cusped magnetic system (0.2 T) along with a sheet electron beam gun (50 mA). Beam-wave interaction simulations utilizing 100 periods demonstrate a peak power of 14 W and continuous frequency tuning from 0.295-0.375 THz for the proposed slow wave structure, whereas the staggered double-vane slow wave structure achieves 8.5 W peak power and frequency tuning from 0.308-0.366 THz. The sensitivity of the output power to the added ridge geometry is also analyzed. These findings indicate that the novel ridge-loaded staggered double vane slow-wave structure is promising for developing high-power broad frequency tunable terahertz radiation sources.

## Introduction

Extensive research has been conducted in recent years on vacuum electronic terahertz (THz) radiation sources owing to their notable advantages such as high power, wide bandwidth, and high efficiency^1–9^. These devices are particularly sought after in various applications like medical imaging and diagnostics, security, and screening, industrial non-destructive testing, etc.^10–22^. Nonetheless, the challenge persists in creating stable, and broad tunable high-power devices, limiting the utilization and advancement of terahertz technology across diverse fields^23^.

Among various THz sources, backward wave oscillators (BWO) have been subject to research to enhance their performance, including improved output power, broad tunability, and efficiency. As the operating frequency increases, the surface roughness of the slow wave structure (SWS) material becomes a significant factor affecting transmission loss and requires careful consideration. In recent years, various novel SWS, including the folded waveguide (FW) and staggered double-vane (SDV) have garnered significant attention due to their inherent planar configuration. Among these, the SDV-SWS, introduced by UC-Davis in 2008^24^, shows considerable promise. Planar configurations lend themselves to straightforward fabrication using micro- and nano-machining techniques such as Computer Numerical Control (CNC)^25–29^. Additionally, the all-metal packaging ensures excellent thermal capacity, contributing to higher output power and gain. At the same time, the waveguide-like form allows for broader operational bandwidth^4^.

As is widely recognized, sheet electron beams (SEB) have the potential to offer significantly high currents due to their horizontal extensibility and minimal space charge fields. By transporting high currents within relatively compact lateral dimensions, they naturally support miniaturization, high operating frequencies, and high output power. However, creating and transporting SEBs pose significant challenges requiring strong magnetic focusing fields. Moreover, achieving long transport distances for the SEB using a uniform magnetic field is generally difficult due to the diocotron instability. Hence, periodic cusped magnet (PCM) fields are employed. Compared to uniform magnetic fields, PCM magnetic fields minimize the instability of SEBs and enable device miniaturization.

A 0.22 THz broadband and high-power SDV traveling-wave tube has been designed and experimentally verified^4^. A 0.22 THz high-power ultra-wideband SDV SWS with double beams is designed and fabricated^30^. Both odd and even modes of the fundamental mode are adopted to align with the electron beam, resulting in a substantial increase in bandwidth. Various staggered double vane slow-wave structures with pencil beam tunnels are designed and fabricated at 0.22 and 0.33 THz, respectively^20^. A shape-optimized SDV SWS with −3dB bandwidth of 50 GHz at 0.22 THz is experimentally investigated^25^. The shape-optimized SDV SWS leverages the benefits of higher interaction impedance, reduced transmission loss, and decreased phase velocity compared to SDV SWS configurations. Table.1 summarizes the findings of the above-mentioned articles.

**Table 1:** Experimental Research on SDV SWS for THz Radiation Sources: A Comparative Overview.

**Year**	**Frequency**	**Type of electron beam**	**Beam voltage**	**Beam current**	**−3 dB bandwidth**
2022	0.22 THz	Sheet	24.6 kV	150 mA	50 GHz
2022	0.22 THz	Circular	20–21 kV	2$\times$80 mA	70 GHz
2021	0.22 THz	Circular	20.6	2$\times$80 mA	70 GHz
2021	0.33 THz	Circular	15.3	40 mA	40 GHz

**Fig. 1:**
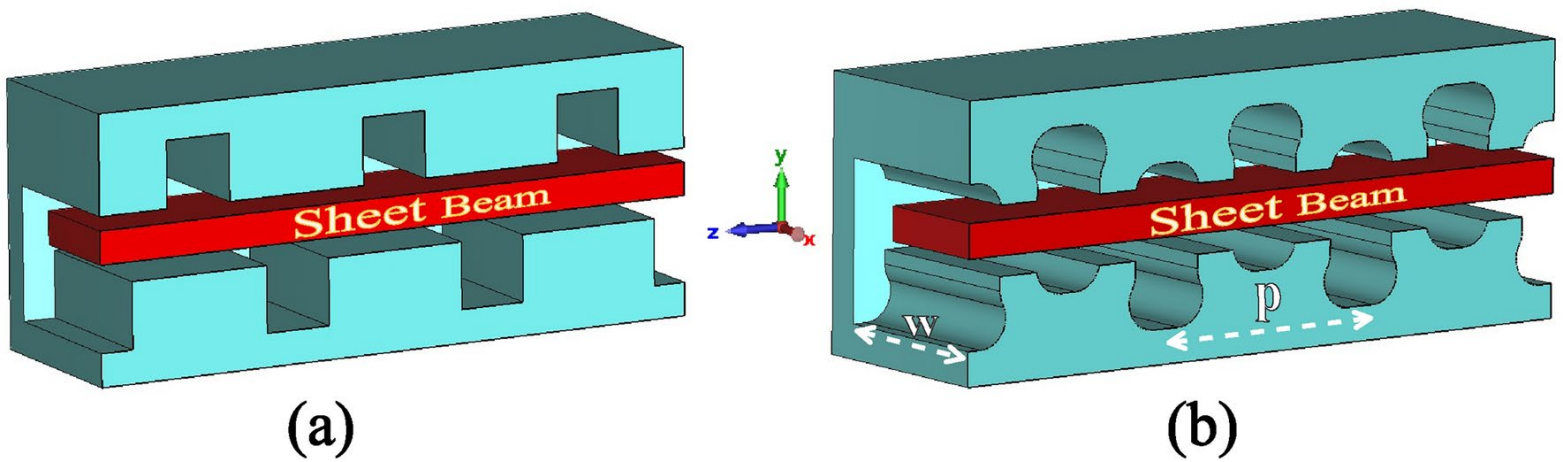
(**a**) Three cells of SDV SWS along with SEB. (**b**) Three cells of RL-SDV SWS along with SEB.

To achieve high output power and broad frequency tunability, a ridge-loaded staggered double vane (RL-SDV) SWS based on T-shaped SDV SWS^31^ for 0.34 THz BWO is proposed as shown in Fig. 1. Enhanced interaction impedance and electric field distribution in the beam-wave interaction region are achieved using a modified shape of the grating teeth. To validate the proposed SWS design, a 0.34 THz BWO is designed. The reflection and transmission characteristics of the BWO through simulation and cold test along with fabrication details are discussed. A PCM system along with a SEB gun is designed to show successful beam transmission over the complete length of the BWO. The beam-wave interaction performance of the RL-SDV SWS is compared with SDV SWS in terms of output power and frequency tunning properties. This section also includes the sensitivity analysis of the RL-SDV SWS-based BWO regarding added ridge geometry. The conclusion section summarizes the major findings of this research.

## High-frequency characteristics of the RL-SDV SWS

### Electric field and energy density distribution

**Fig. 2:**
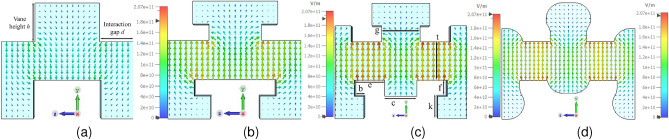
(**a**) Electric field in SDV SWS. (**b**) Electric field in T-shaped SDV SWS. (**c**) Electric field in RL-SDV SWS. (**d**) Electric field in machinable blended RL-SDV SWS.

Fig. 2shows the simulated electric field distribution results in a single cell of SDV SWS and RL-SDV SWS obtained using CST Microwave Studio. This configuration generates a strong, symmetric axial electric field along the electron beam’s path^32^. Increasing the height *h* of the vanes or reducing the interaction gap *d* near the top of the vanes increases the values of surface charge and electric field. Increasing the value of *h*, also enlarges the volume of the cavities above and below the beam tunnels, thereby enhancing the electromagnetic (EM) field distribution within the cavity^33^. The height of the vanes cannot be increased beyond a certain limit. If *h* is increased towards the SEB tunnel, it will reduce the space available for the SEB transport thus posing the problem of particle collision with the SEB tunnel. If *h* is increased towards the outer side by increasing the depth, the transmission and reflection characteristics of the device get disturbed greatly. Thus, the ridges are incorporated at the top of the vanes to decrease the interaction gap *d* while keeping the vane height constant. The maximum electric field for the SDV SWS can reach up to $1.82\times 10^{11} Vm^{-1}$ 2a. The electric field is enhanced for the T-shaped SDV SWS as shown by Fig. 2b and its maximum value has now reached up to $1.91\times 10^{11} Vm^{-1}$. The addition of another ridge in the middle of the T-shaped vane further enhances the electric field to a maximum value of $2.07\times 10^{11} Vm^{-1}$ as shown by Fig. 2c. Due to the miniaturized nature of the SWS and the machining limitations of the computer numerical control (CNC) machine, the internal corners of the SWS are blended by a radius of 50$\mu$m. This blending has a negligible effect on the maximum value of the electric field within SWS ($2.03\times 10^{11} Vm^{-1}$) as shown in Fig. 2d. This enhanced electric field distribution along with increased longitudinal electric field $E_z$(discussed later) is the most important factor in increasing the interaction impedance and beam-wave interaction thus increasing the output power^34^.

**Fig. 3:**
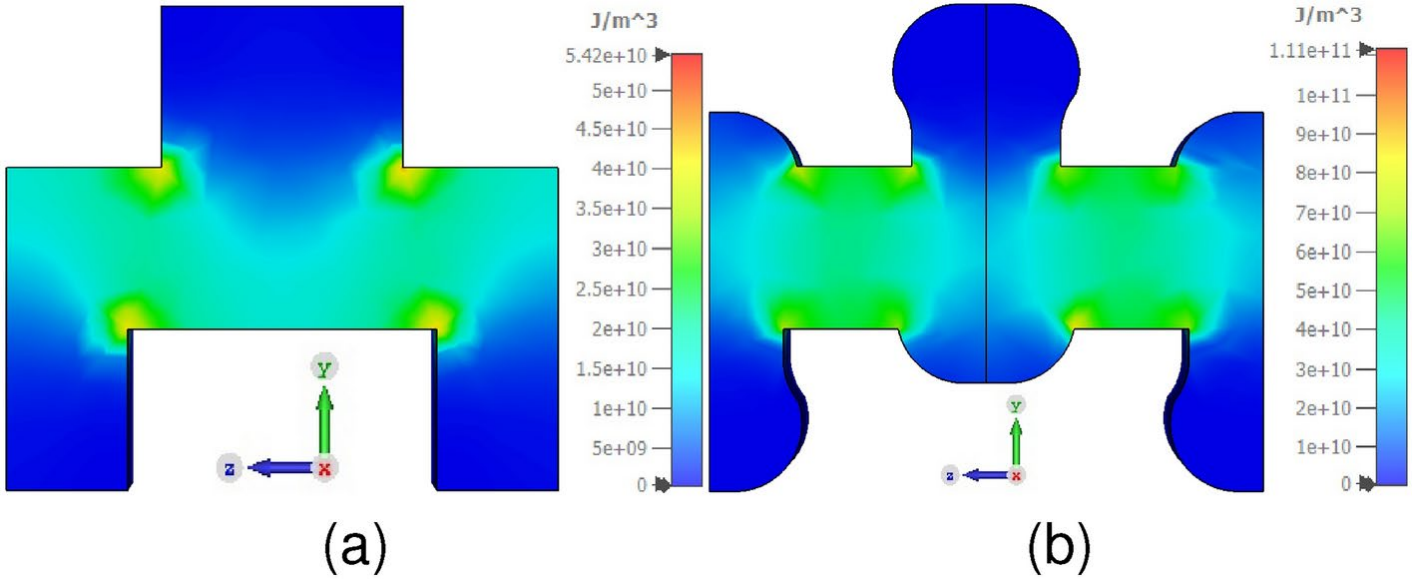
(**a**) Energy density in the SDV SWS. (**b**) Energy density in RL-SDV SWS.

Simulations are performed to plot the energy density within the interaction region of SWS to investigate the effects of the addition of ridges in the SWS further. The energy density within the SWS represents the concentration of the EM energy carried by the EM waves. This energy density increases as the EM wave is slowed down in the SWS. Fig. 3 shows the comparison of energy density in the SDV and RL-SDV SWS. The maximum energy density distribution in RL-SDV SWS has almost doubled thus increasing the beam-wave interaction.

### Dispersion characteristics

**Fig. 4:**
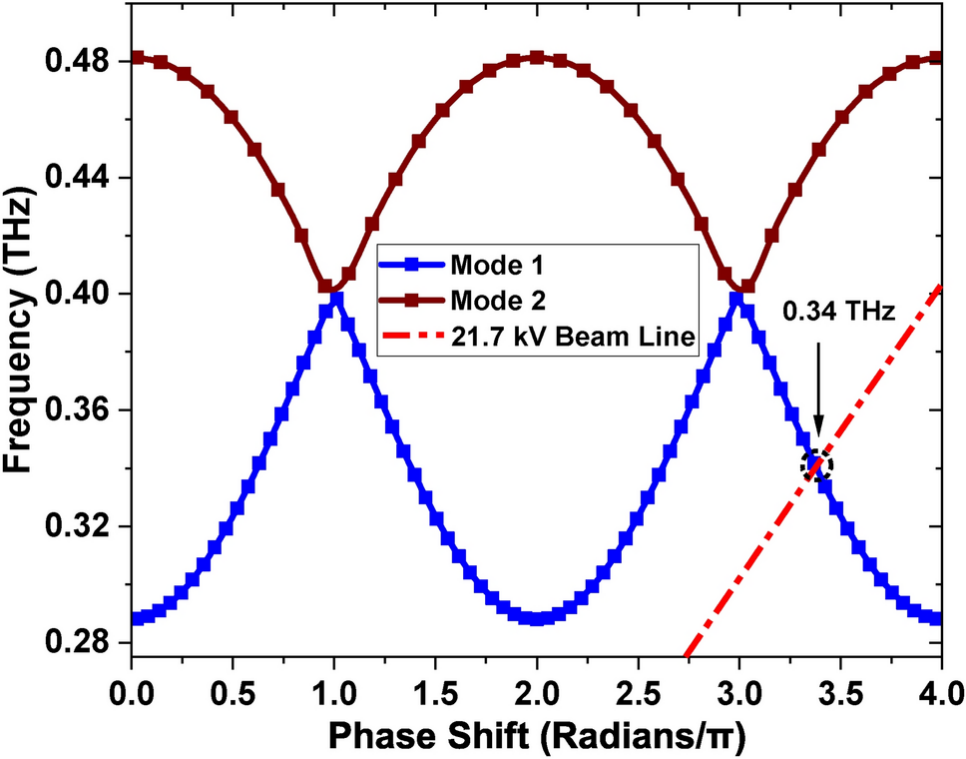
Dispersion characteristics of the SDV & RL-SDV SWS along with 21.7 kV beam line.

Generally, the interaction impedance tends to decrease with an increase in the operating mode and the number of spatial harmonic^25^. Consequently, the RL-SDV SWS primarily operates in the fundamental mode. Despite having the same passband, the dispersion significantly varies among the different spatial harmonics of the fundamental mode. Because of the symmetry of the RL-SDV SWS, the EM field dispersion equation solutions can be categorized into odd and even modes. Both mode 1 and mode 2 are considered fundamental modes. The mode frequencies for a phase shift of 0–4$\pi$ radians are plotted in Fig. 4. The 21.7 kV beam line intersects the odd mode 1 of dispersion curves at an operating frequency of 0.34 THz. While lower spatial harmonics provide better interaction impedance and higher output power, they require impractically high voltages (more than 200 kV) for the miniaturized SWS. Thus, the *n = −2* spatial harmonic is selected for operation.

The high-frequency properties of the SWS are greatly impacted by geometric parameters such as *w, p, t, g*, and *f* requiring extensive simulations for optimization. While maintaining the values of the other variables constant, the effect of each variable on the dispersion is analyzed using simulations. Table 2shows the optimized key dimensional parameters of the RL-SDV SWS. The dimensions of the SWS are chosen to maximize the frequency passband of the odd mode 1 to increase the frequency tunability range of the BWO^33^.

**Table 2:** Dimensional Parameters of the RL-SDV SWS.

Parameter	Value (mm)	Parameter	Value (mm)	Parameter	Value (mm)
**w**	0.52	**t**	0.12	**p**	0.41
**e**	0.095	**c**	0.10	**g**	0.12
**f**	0.05	**b**	0.03	**k**	0.07

### Interaction impedance

**Fig. 5:**
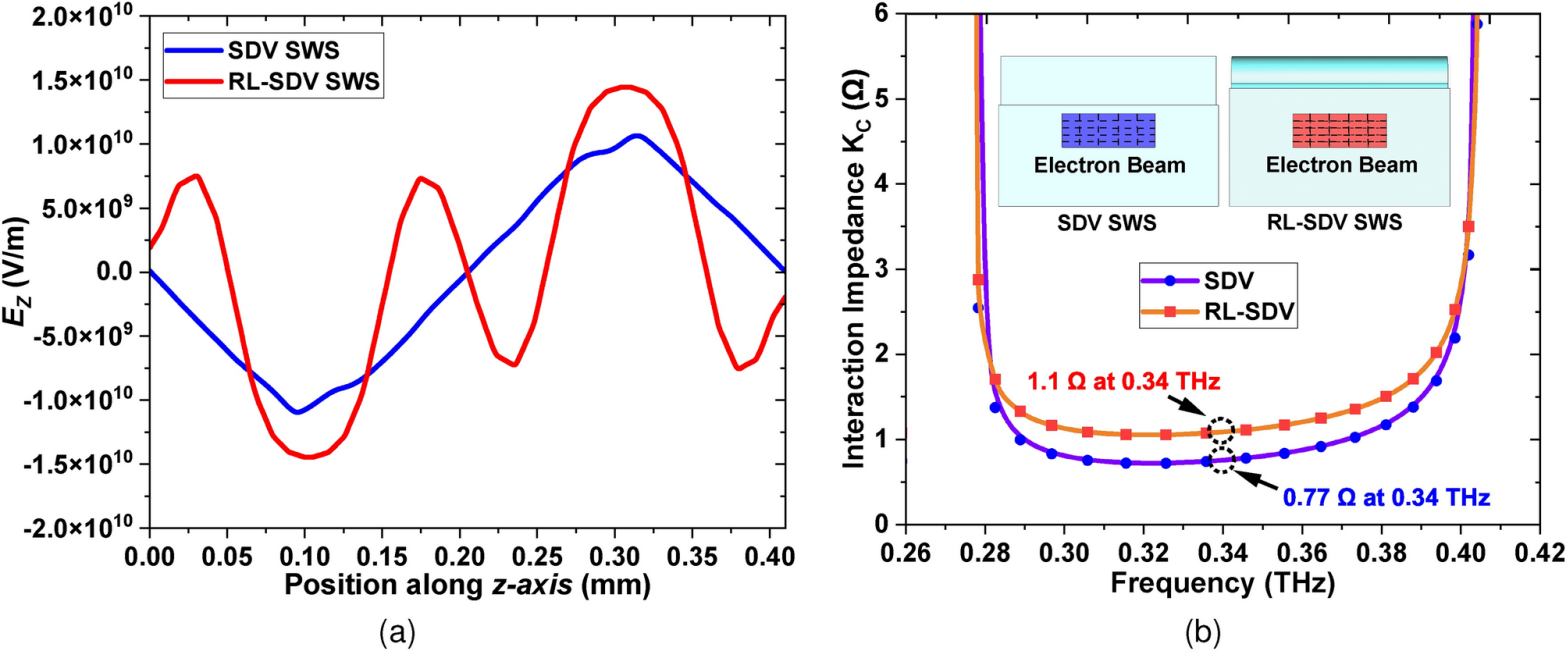
(**a**) Distribution of axial electric field $E_z$ along the beam tunnel center of both SDV and RL-SDV SWS. (**b**) Comparison of average interaction impedance of the SDV and RL-SDV SWS.

The interaction impedance determines the strength of the interaction between the electron beam and the EM waves. A higher interaction impedance generally leads to greater wave slowing, which prolongs the interaction time between the beam and the wave, leading to higher output power. It is mathematically defined as:

1 $$\begin{aligned} K_c=\frac{|E_{zn}|^2}{2{\beta _n}^2P_w} \end{aligned}$$

where $E_{zn}$ is the amplitude of the electric field component $E_z$ of *nth* spatial harmonic, $\beta _n$ is the *nth* spatial harmonic’s phase constant and $P_w$ is the power flow along *z* direction. Thus we can see that $E_z$is a key parameter directly affecting the interaction impedance of the SWS^9^. The electric field component $E_z$ for the *n = −2* spatial harmonic is plotted in Fig. 5a for both the SDV and RL-SDV SWS. These values are plotted at the same position along the center of the beam tunnel for both SWS. It can be seen that $E_z$ is significantly increased for the proposed RL-SDV SWS which in turn increases the interaction impedance.

Fig. 5b shows a comparison of the interaction impedance of both the SWS obtained using High-Frequency Simulation Software (HFSS). The interaction impedance is calculated at different positions on a $6\times 6$ grid on the entire area of the SEB tunnel for a phase shift of 3$\pi$−4$\pi$ radians corresponding to *n = −2* spatial harmonic. An average value is then plotted for a broad frequency range (0.26 - 0.42 THz). It can be seen that the average interaction impedance at 0.34 THz in the RL-SDV SWS has increased significantly (1.1 $\Omega$) as compared to the SDV SWS (0.77 $\Omega$). This increased interaction impedance implies a stronger coupling between the electron beam and the EM waves thus increasing output power.

## Transmission characteristics of the RL-SDV SWS

### Simulation of the BWO circuit

**Fig. 6:**
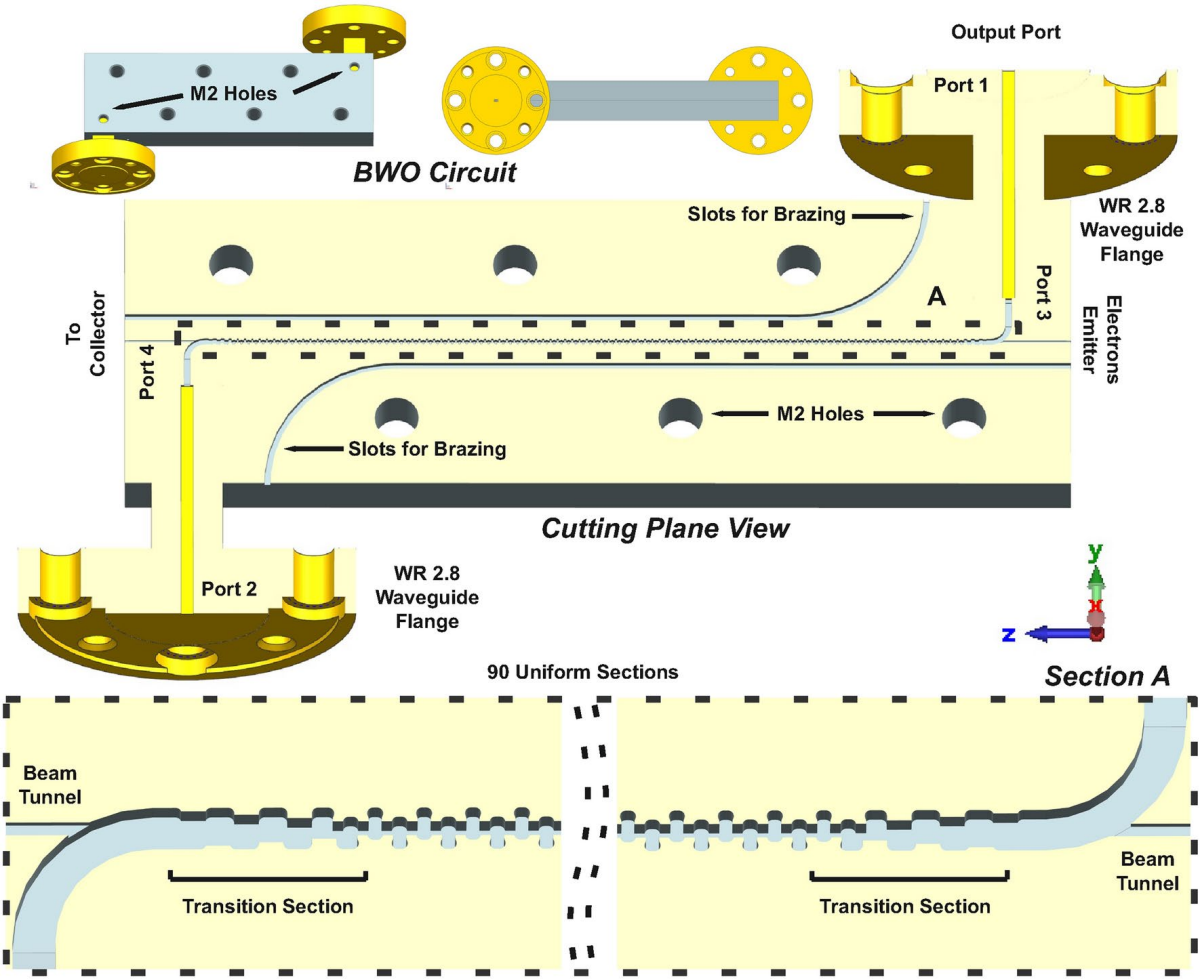
BWO circuit simulation and fabrication model along with beam tunnel and WR 2.8 waveguide flanges.

To examine the transmission and reflection characteristics of the RL-SDV SWS, a simulation model comprising WR 2.8 waveguide, transition sections, and uniform sections, is constructed as depicted in Fig. 6. The transition section facilitates impedance matching between the uniform section and the rectangular waveguide WR 2.8. The transition section is implemented by gradually trimming the top and bottom of the vanes at both ends of the SWS. The total number of periods for the uniform and transition sections is 100, resulting in a total transmission model length of approximately 46 mm (with the beam tunnel). The dimensions of the beam tunnel are carefully chosen (0.32 mm $\times$0.12 mm) to prevent the leakage of electromagnetic waves through ports 3 and 4. The cutoff frequency for the beam tunnel is approximately 0.47 THz. In the simulations, the material surface roughness of the oxygen-free high-conductivity (OFHC) copper is accounted for by incorporating the effective conductivity, computed as follows^35^:

2 $$\begin{aligned} \sigma _{eff}=\sigma _0[1+\frac{2}{\pi }arctan{1.4(\frac{Ra}{\delta })^2}]^{-2} \end{aligned}$$

Here, $\sigma _0 = 5.8\times 10^7$ $Sm^{-1}$ represents the material conductivity of OFHC copper having an ideally flat surface, $\sigma _{eff}$ is the effective conductivity of rough surface, $\delta =(\mu _0\omega \sigma _0)^{\frac{-1}{2}}$ denotes the skin depth, and *Ra* signifies the surface roughness measured in $\mu m$. As indicated by 2, the effective conductivity will decrease with increasing surface roughness or frequency. The measured surface roughness of the fabricated SWS is approximately 0.07 $\mu m$. The surface roughness is measured using the VR-5000 3D Optical Profiler. This profiler emits structured light through several double-telecentric lenses to minimize distortion. Height differences in the copper surface cause distortions in the light bands, which are then converted to height values using triangulation techniques. The effective conductivity of the background material (copper) is therefore set to $2.2\times 10^{7}$ $Sm^{-1}$ to take into account the surface roughness effects.

### Fabrication and cold test

The nano-CNC high-speed milling machine (GF Mikron HSM 500) known for its precision capabilities is used for SWS fabrication. The shape and position accuracy of the machine used is 5 $\mu m$ and 2 $\mu m$ respectively, with a minimum surface roughness of approximately 0.07 $\mu m$ in copper. For ease of the fabrication process, the SWS is divided into two identical halves along the centerline of the E-plane for milling. Oxygen-free copper (OFC) is used in manufacturing the 2 SWS halves while the WR 2.8 flanges are made from brass. The machined components have a minimum corner radius of 50 $\mu$m. Manufacturing of the SWS employs a milling tool with a cutting diameter of 0.1 mm, an under-neck length of 0.5 mm, and a spindle speed of 42,000 rpm. The fabrication process took approximately 60 hours to complete a pair of SWS and flanges.

**Fig. 7:**
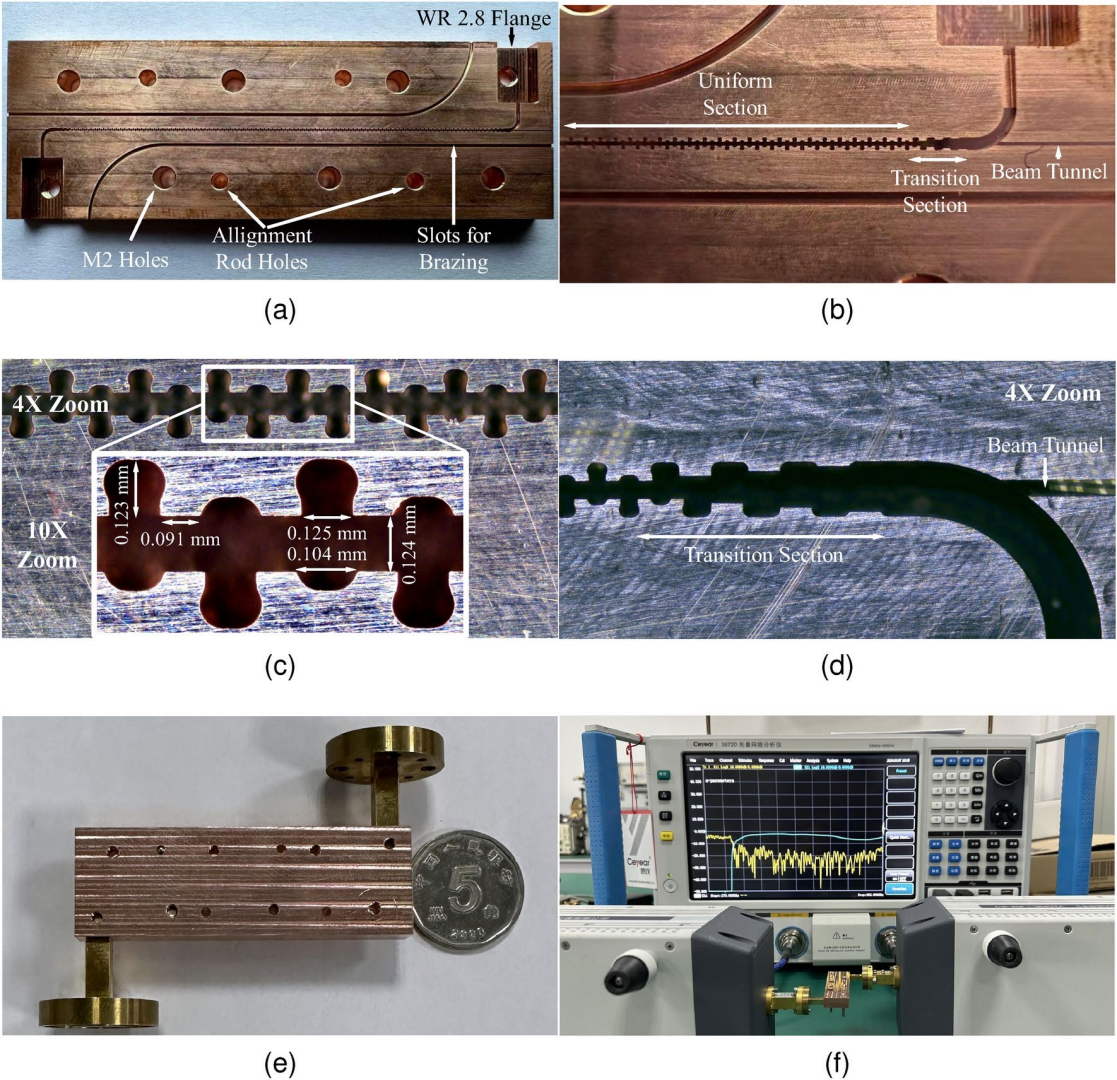
(**a**) One half of the RL-SDV SWS. (**b**) Zoomed-in view of the RL-SDV SWS. (**c**) Microscopic view of uniform sections. (**d**) Microscopic view of the transition section and beam tunnel. (**e**) Complete assembly view. (**f**) Experimental platform for cold test.

Fig. 7a and 7b show one fabricated half of the SWS. Four alignment rods placed in alignment holes serve the purpose of precise alignment of the two fabricated SWS halves. There are slots (0.35 mm $\times$ 0.35 mm) left for the brazing of the two halves together later to minimize the misalignment risks. Fig. 7c and 7d show the optical microscopic views of the uniform sections, transition sections, beam tunnel, and measured dimensions. There is very little deviation between the designed values and measured values with a tolerance of approximately ±5 $\mu m$. The SWS parts, milled using nano-CNC technology, undergo degreasing in ultrasonic cleaners to eliminate organic residues and copper debris generated during milling. Acid treatment is employed to eliminate surface oxides. Both halves of the RL-SDV SWS are assembled along with flanges using M2 screws as shown in Fig. 7e. The transmission and reflection characteristics test platform, as depicted in Fig. 7f, is established. A Vector Network Analyzer (VNA) along with frequency extenders with the frequency range of 0.26 - 0.4 THz is used.

**Fig. 8:**
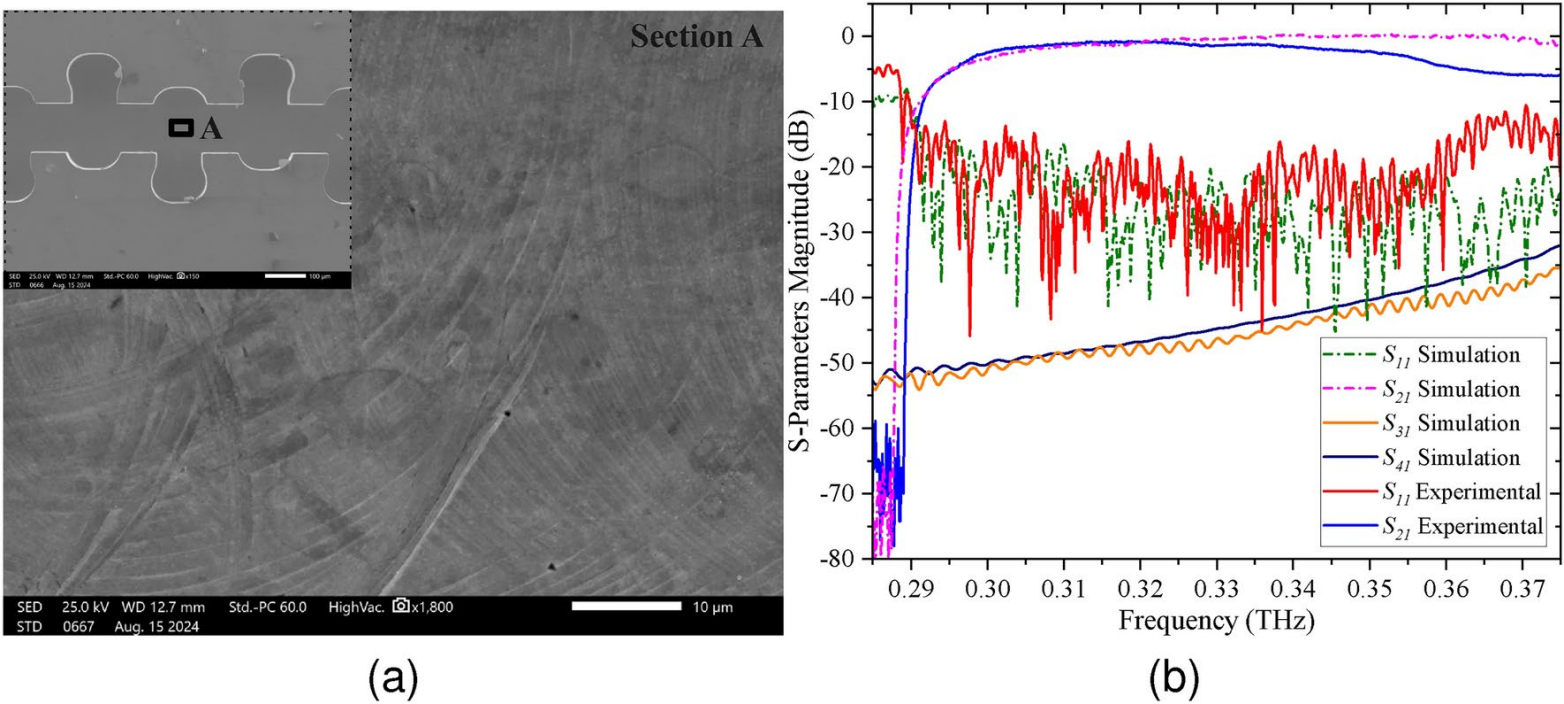
(**a**) Scanning Electron Microscope (JEOL JSM-IT500LV) images of the SWS showing surface finishing. (**b**) Measured and simulated S-parameters for the fabricated RL-SDV SWS.

Fig. 8a shows the scanning electron microscope (SEM) images (JEOL JSM-IT500LV) providing a detailed view (Section A) of the surface morphology of the fabricated SWS. The SEM images reveal a relatively smooth surface finish, essential for minimizing electron scattering and ensuring optimal device performance. The precision in the fabrication process is evident from the uniformity of the ridges and the absence of significant surface defects or irregularities. Any minor imperfections observed are within acceptable limits and do not compromise the overall functionality of the structure.

Fig. 8b shows the comparison between measured and simulated S-parameters for the designed RL-SDV SWS for a frequency range of 0.285–0.375 THz. The measured $S_{21}$ and $S_{11}$ are approximately above −2 dB and below −17 dB respectively at 0.34 THz. The simulated $S_{21}$ and $S_{11}$ are approximately above −0.5 dB and below −20 dB respectively for the same frequency. Similarly, the simulated $S_{31}$ and $S_{41}$ are well below −40 dB at 0.34 THz. The measured results agree well with the simulated results at lower frequencies. However, the difference between measured and simulated results becomes significant at higher frequencies due to several reasons. Minor deviations in the structure’s geometry due to fabrication tolerances can significantly impact performance at higher frequencies. Additionally, errors during coupling of the SWS halves, WR 2.8 flanges, and VNA frequency extender flanges disturb the transmission and reflection characteristics at higher frequencies.

## Electron Optical System (EOS)

**Fig. 9:**
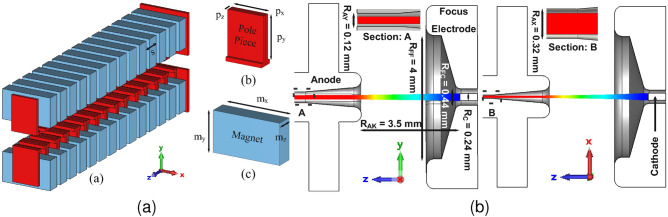
(**a**) Schematic of the proposed PCM. ($p_x$ = 4.6 mm, $p_y$ = 6 mm, $p_z$ = 1.13 mm, $m_x$ = 8.6 mm, $m_y$ = 5 mm, $m_z$ = 2.37 mm, *s* = 3.5 mm). (**b**) 3D model of the sheet electron beam gun along with particle trajectory in *yoz* plane and *xoz* plane.

Using a uniform magnetic field to focus a SEB over long distances is challenging due to “diocotron” instability and the need for large magnets, hindering THz device miniaturization. The periodic magnetic field from PCM effectively suppresses this instability and aids miniaturization. The PCM system includes pole pieces and permanent magnets as shown in Fig. 9a along with geometrical dimensions. To focus a 21.7 kV, 50 mA electron beam, a Brillouin magnetic field of approximately 0.2 T is required. The magnetic field generated by the PCM system is imported into the particle tracking solver to simulate the SEB gun. As the operating frequency increases into the terahertz range, vacuum electron devices require significantly higher minimum current densities, often reaching hundreds of A/cm² as in this case. Despite extensive research, these high current densities remain a challenge. Thermionic cathodes are an affordable and mature technology, but they face limitations like high operating temperatures, thermal dissipation, large volume, slow response times, etc. In contrast, field electron emission carbon nanotube (CNT) cold cathodes offer near-instantaneous turn-on, compact designs, minimal working volume, and room-temperature operation, making them a promising alternative^36–38^.

In this article, the field emission model is used to simulate the electron gun using a $Ni_{80}Cr_{20}$ alloy. The Fowler-Nordheim equation for the current density is given by:

3 $$\begin{aligned} J=AE^2e^{(-B/E)} \end{aligned}$$

The 21.7 kV beam voltage applied to an anode-cathode gap of 3.5 mm, results in E = 6.2 $MVm^{-1}$. The values of A and B are approximately $1.9\times 10^{-4}$ $AV^{-2}$ and $6.36\times 10^7$ $Vm^{-1}$ respectively. This results in an emission current density of approximately 25.6 $Acm^{-2}$ at the cathode surface. When the electron beam enters the SWS, the current density reaches up to 260 $Acm^{-2}$.

Velocity and energy spread in electron beams can significantly impact the performance and accuracy of electron gun simulations. Variations in velocity and energy can lead to beam divergence, reduced focus, and increased scattering, which in turn affect the beam’s interaction with the electromagnetic fields in devices. Ignoring these factors in simulations may lead to idealized results, while considering them provides a more realistic representation, improving the accuracy of predictions for beam dynamics and device performance. Therefore a velocity spread of 5% is considered in the field emission model of particle tracking solver for a more realistic non-ideal behavior.

**Fig. 10:**
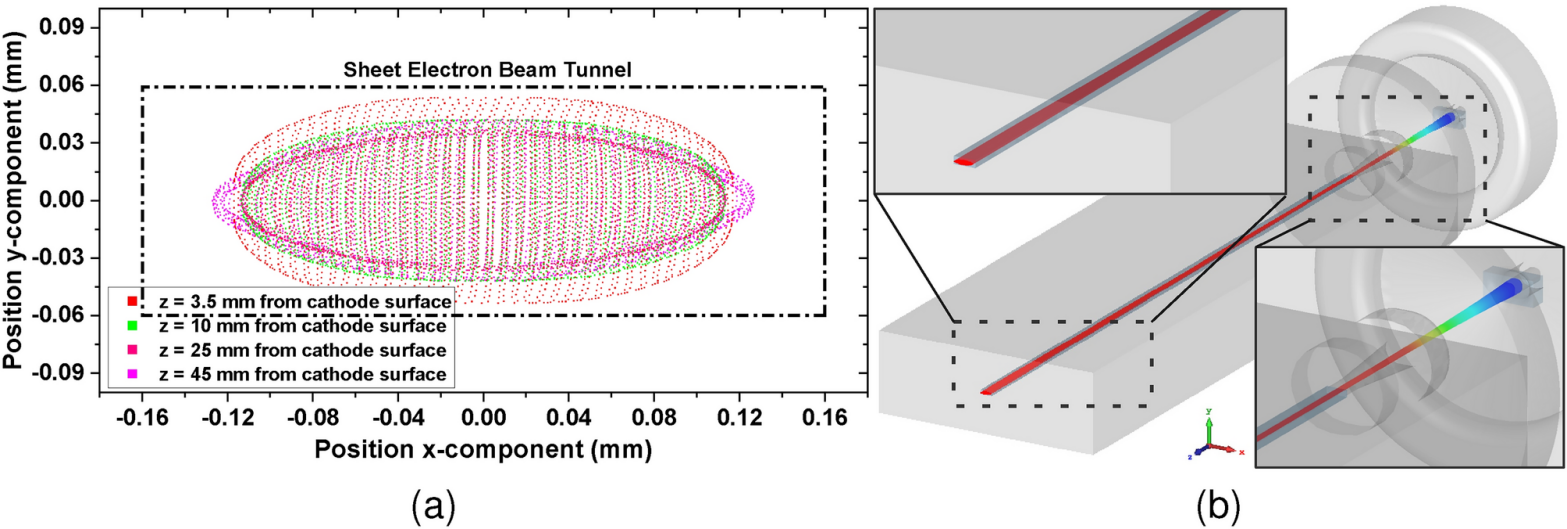
(**a**) Sheet electron beam envelope at different positions along the beam tunnel.(**b**) Beam trajectory of the SEB gun through the SWS.

**Fig. 11:**
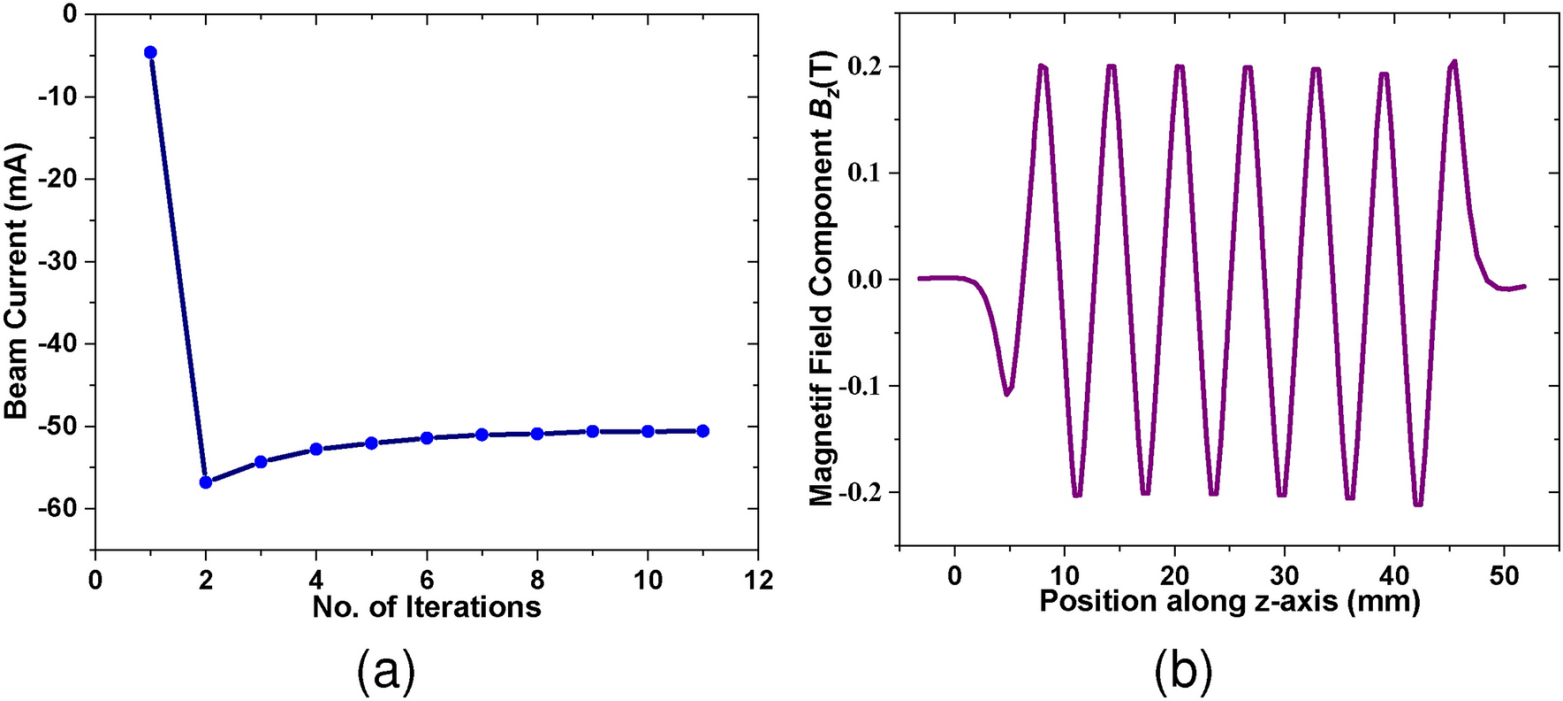
(**a**) Emission current of the SEB gun (**b**) Distribution of magnetic field component $B_z$ along z-axis.

Fig. 9b shows the SEB gun along with particle trajectories in both *yoz* and *xoz* planes. The cathode and the focusing electrodes are set to an equipotential of −21.7 kV, while the anode is grounded at 0 V. The anode tunnel is designed to have dimensions equivalent to the beam tunnel. Due to the rectangular curvature of the anode tunnel, the SEB is compressed more in the *yoz* plane than in the *xoz* plane. Fig. 10a shows the beam envelope at different positions along the beam tunnel. Fig. 10b shows the three-dimensional trajectory of the beam through the SWS. It can be seen that a transmission rate of 100$\%$ is achieved with an acceptable beam shape and stiffness. Fig. 11a displays the emitted SEB current. Fig. 11b shows the magnetic field distribution $B_z$ along the z-axis, peaking at 0.2 T.

## Beam-wave interaction of the RL-SDV SWS

The investigation into beam-wave interaction utilizes PIC simulations conducted through CST Particle Studio. As the PIC simulations in the terahertz frequency band take a lot of time, an emitter is used on one end of the SWS in place of the whole electron gun using the DC emission model of PIC. A constant magnetic field $B_z$ of 0.2 T is used to focus the beam. A rectangular emitter measuring 0.24 mm $\times$ 0.08 mm in cross-sectional size is utilized. The beam operates at a voltage of 21.7 kV and a current of 50 mA. This configuration results in a beam current density of 260 A$cm^{-2}$which is quite achievable using carbon nanotube cold cathodes^39^. The DC emission model also uses a velocity spread of 5% just like the field emission model used in the previous section. In short, the EOS section uses a different emission model and solver to simulate the electron gun along with the periodic cusped magnetic system to validate the high current density sheet electron beam transmission through the complete length of the BWO circuit. The PIC simulations use the DC emission model (using the same beam voltage, current, velocity spread, beam, and tunnel dimensions) to effectively predict the beam-wave interaction.

**Fig. 12:**
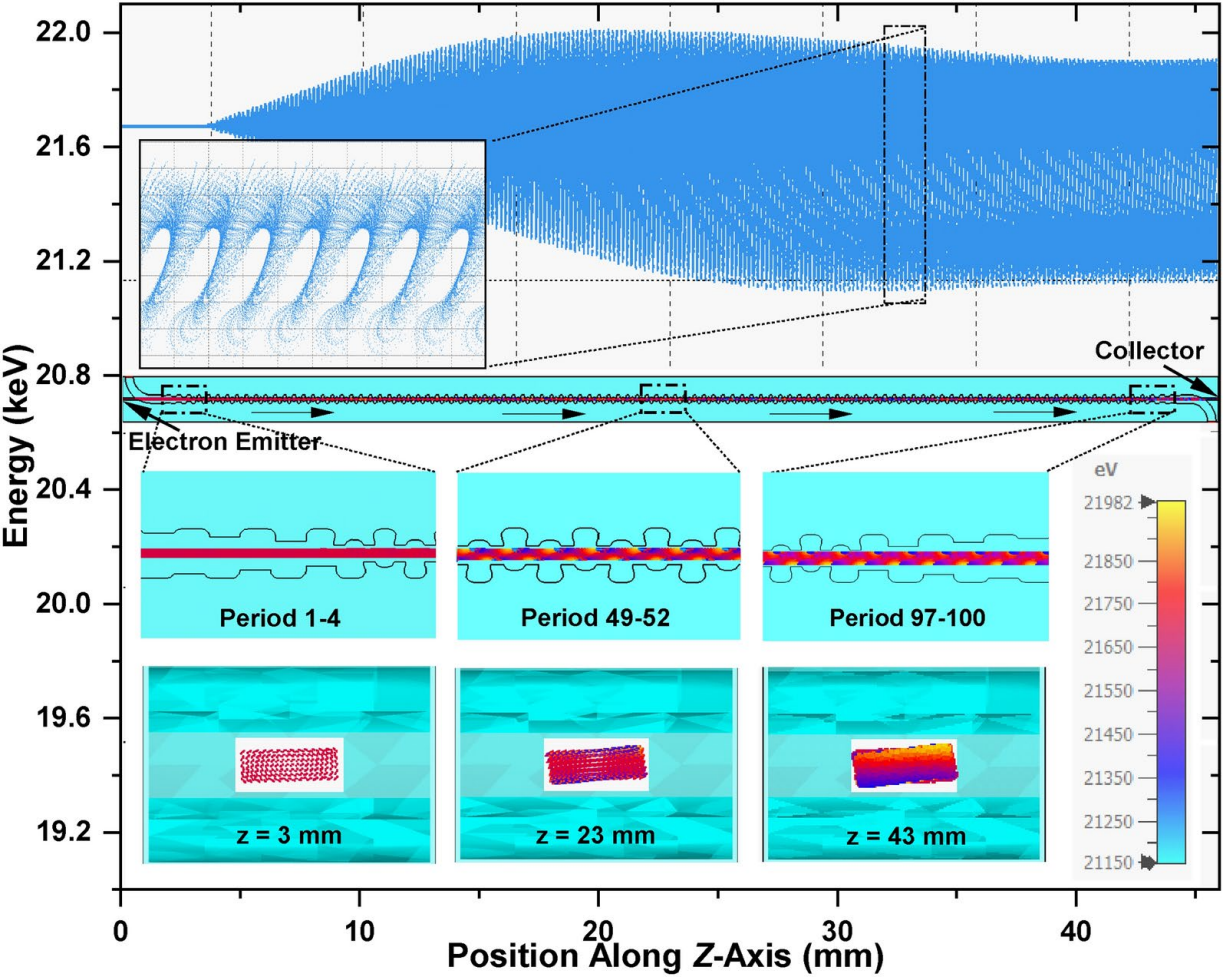
Energy phase space diagram of the SEB at 30 ns along with SEB trajectory at different positions along *z-axis*.

Port 1 is configured as the output port where the BWO output power is measured. Port 2 is treated as a matched load to absorb any residual power and prevent reflections back into the system. Fig. 12 depicts the simulation outcomes of the SEB’s energy phase space diagram at 30 ns along with SEB trajectory at different positions along the tunnel. The results indicate that the electrons expend more energy than they acquire suggesting a transfer of energy from the beam to the EM waves.

**Fig. 13:**
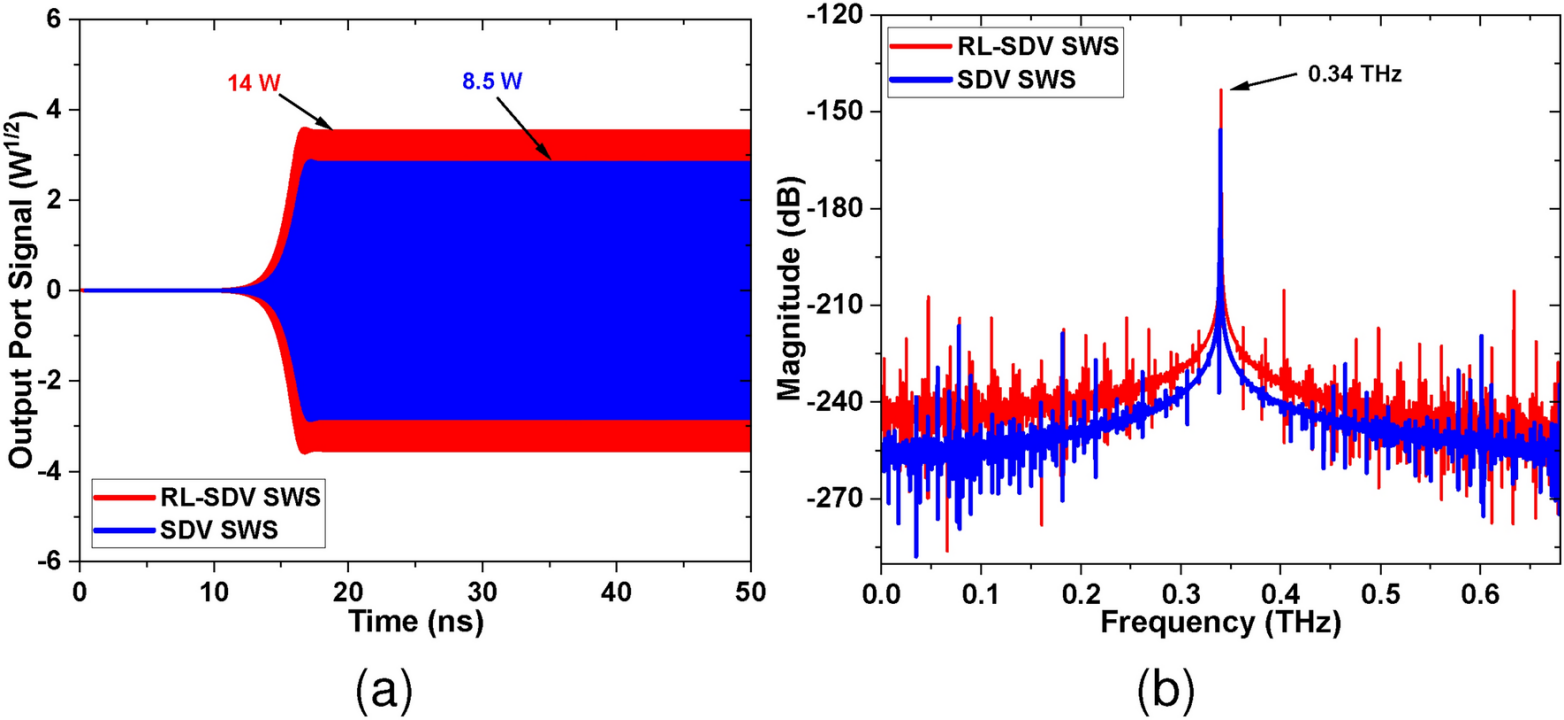
(**a**) Output port signal vs time for both SWS. (**b**) The frequency spectrum of both SWS.

Fig. 13a shows the output port signal for both the SDV and RL-SDV SWS BWOs plotted against time. The plot shows stable output port signals for both designs. The output port mode in the rectangular waveguide is $TE_{10}$. The calculated output power for RL-SDV SWS-based BWO is approximately 14 W as compared to 8.5 W for the SDV SWS-based BWO. Fig. 13b shows the frequency spectra for both the BWOs showing operating frequency at 0.34 THz. The higher harmonics are notably minimal, signifying the purity of the output signal.

**Fig. 14:**
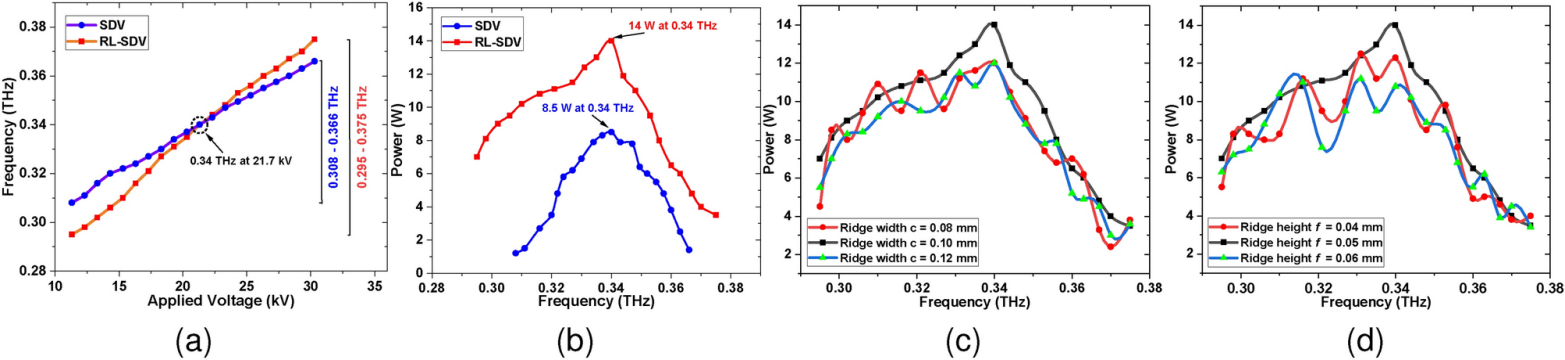
(**a**) Frequency vs voltage plot for both SWS. (**b**) Power vs frequency plot for both SWS. (**c**) Sensitivity analysis of the output power versus frequency with ridge width *c* = 0.1 ± 0.02 mm. (**d**) Sensitivity analysis of the output power versus frequency with ridge height *f* = 0.05 ± 0.01 mm.

Fig. 14a and 14b show the tunning properties for both the BWOs. With the current kept constant at 50 mA, the voltage is varied from 11 kV to 30 kV. For the SDV SWS-based BWO, the output frequency increases uniformly from 0.308–0.366 THz. The frequency of the RL-SDV SWS-based BWO increases uniformly from 0.295–0.375 THz. At 0.34 THz, both devices operate at maximum output peak power. The sensitivity of the PIC analysis concerning the ridge width *c* and ridge height *f* is also analyzed as these dimensions are subjected to machining tolerances, which may have adverse effects on device performance. Fig. 14c and 14d illustrate the PIC sensitivity analysis of output power versus frequency for variations in ridge width *c* and ridge height *f*. There is a significant drop in power with changes in these dimensions. These differences are explained by the fact that the synchronous condition of the beam-wave interaction gets disturbed due to variations in these dimensions. Therefore, the geometric machining tolerances of these key parameters should be limited to less than or equal to ±5 $\mu$m when the BWO is operating at a fixed beam voltage.

## Conclusion

In conclusion, the RL-SDV SWS presents promising advancements for THz radiation sources. The modifications yield enhanced electric field distribution and improved interaction impedance within the beam-wave interaction region compared to SDV SWS designs. Through comparative analysis, it has been demonstrated that the proposed RL-SDV SWS achieves superior beam-wave interaction characteristics, with peak power reaching 14 W, surpassing the performance of the SDV SWS with 8.5 W peak power. Steady transmission of the sheet electron beam (21.7 kV and 50 mA) is achieved by using a magnetic field of 0.2 T. Experimental validation through fabrication and cold testing of RL-SDV SWS further corroborates these findings, revealing satisfactory performance metrics such as $S_{21}$ above −2 dB and $S_{11}$ below −17 dB for 100 periods of the RL-SDV SWS. PCM system is designed to show the transmission of the SEB through the BWO. The sensitivity of the output power to the ridge geometry is also analyzed. These results underscore the potential of the RL-SDV SWS in the development of broad-frequency terahertz sources where different frequencies are required at different times and frequency agility is beneficial.

## Data Availability

The datasets used and/or analysed during the current study available from the Mr. J.L. author on reasonable request.
